# Increased activation product of complement 4 protein in plasma of individuals with schizophrenia

**DOI:** 10.1038/s41398-021-01583-5

**Published:** 2021-09-22

**Authors:** Agnieszka Kalinowski, Joanna Liliental, Lauren A. Anker, Omer Linkovski, Collin Culbertson, Jacob N. Hall, Reenal Pattni, Chiara Sabatti, Douglas Noordsy, Joachim F. Hallmayer, Elizabeth D. Mellins, Jacob S. Ballon, Ruth O’Hara, Douglas F. Levinson, Alexander E. Urban

**Affiliations:** 1 Department of Psychiatry and Behavioral Sciences, Stanford University School of Medicine, Stanford, CA 94305 USA; 2 Sierra Pacific Mental Illness Research Education and Clinical Center (MIRECC), VA Palo Alto Health Care System, Palo Alto, CA USA; 3 Translational Applications Service Center, Stanford University School of Medicine, Stanford, CA 94305 USA; 4 Translational Research and Applied Medicine, Stanford University School of Medicine, Stanford, CA 94305 USA; 5 Department of Medicine, Stanford University School of Medicine, Stanford, CA 94305 USA; 6 Department of Psychology, Bar-Ilan University, Ramat-Gan, Israel; 7 Department of Neurology, Stanford University School of Medicine, Stanford, CA 94305 USA; 8 The Neurology Center of Southern California, Temecula, CA 92592 USA; 9 Department of Genetics, Stanford University School of Medicine, Stanford, CA 94305 USA; 10 Department of Biomedical Data Science and Statistics, Stanford University School of Medicine, Stanford, CA 94305 USA; 11 Department of Pediatrics, Stanford Program in Immunology, Stanford University School of Medicine, Stanford, CA 94305 USA

**Keywords:** Schizophrenia, Clinical genetics

## Abstract

Structural variation in the complement 4 gene (*C4*) confers genetic risk for schizophrenia. The variation includes numbers of the increased *C4A* copy number, which predicts increased C4A mRNA expression. C4-anaphylatoxin (C4-ana) is a C4 protein fragment released upon C4 protein activation that has the potential to change the blood–brain barrier (BBB). We hypothesized that elevated plasma levels of C4-ana occur in individuals with schizophrenia (iSCZ). Blood was collected from 15 iSCZ with illness duration < 5 years and from 14 healthy controls (HC). Plasma C4-ana was measured by radioimmunoassay. Other complement activation products C3-ana, C5-ana, and terminal complement complex (TCC) were also measured. Digital-droplet PCR was used to determine C4 gene structural variation state. Recombinant C4-ana was added to primary brain endothelial cells (BEC) and permeability was measured in vitro. C4-ana concentration was elevated in plasma from iSCZ compared to HC (mean = 654 ± 16 ng/mL, 557 ± 94 respectively, *p* = 0.01). The patients also carried more copies of the *C4AL* gene and demonstrated a positive correlation between plasma C4-ana concentrations and *C4A* gene copy number. Furthermore, C4-ana increased the permeability of a monolayer of BEC in vitro. Our findings are consistent with a specific role for C4A protein in schizophrenia and raise the possibility that its activation product, C4-ana, increases BBB permeability. Exploratory analyses suggest the novel hypothesis that the relationship between C4-ana levels and C4A gene copy number could also be altered in iSCZ, suggesting an interaction with unknown genetic and/or environmental risk factors.

## Introduction

**Fig. 1:**
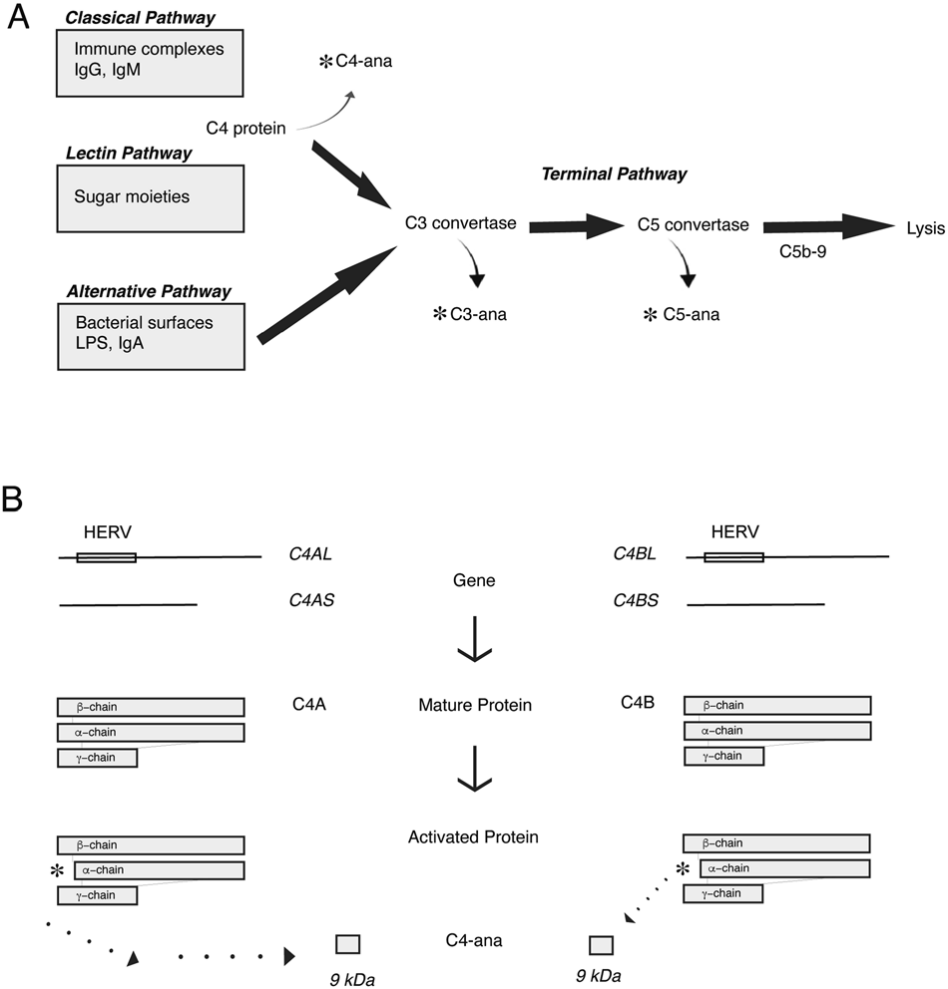
C4A/B genes produce C4A/B protein, which both release C4-ana when activated. **A** C4-ana is produced when C4 protein is activated in the Classical and Lectin Complement Pathways. Similarly, C3-ana is released when C3-convertase is activated and C5-ana is produced upon activation of the Terminal Complement Pathway. **B** An inactive retrovirus, HERV, present between exons 9 and 10 of the C4A or C4B gene, differentiates the long form of the gene from the short form of the gene. The C4A gene produces the C4A (acidic) protein while the C4B gene produces the C4B (basic) protein, differentiated by 5 amino acids as a result of four nucleotide polymorphisms. Both forms of the protein lead to the cleavage product, C4-ana when activated by binding to protein binding partners.

The complement system, a part of the innate immune system, contributes to the first line of defense against pathogens (Fig. 1A). In addition, complement in the brain influences neuronal development and refinement [1]. Dysregulation of complement has been reported in patients with schizophrenia, based on genetic, transcriptomic, and functional studies [2–6].

Genome-wide association studies (GWAS) found an association between schizophrenia and structural variants in the Complement component 4 (C4) gene [7]. C4A and C4B genes have variable gene copy numbers (GCN), and each gene has long (L) and short (S) forms (Fig. 1B). Schizophrenia patients carry more copies of C4A genes and more of the C4AL form, accounting at least in part for the GWAS signal [7]. Mice with higher C4 GCN and increased C4 protein levels show aberrant neuronal development, including increased pruning, accompanied by schizophrenia-like behavioral traits [7–9]. Also linked to schizophrenia is a common intronic allele of the *CSMD1* gene [3, 10], which encodes a complement inhibitor expressed in neural tissue, including developing neurons [11, 12]. CSMD1 deletion in mice leads to neurocognitive deficits [11]. Loss of *CSMD1* expression in human stem-cell-derived neurons increases their susceptibility to activated complement, leading to increased complement deposition on neurites [12].

Changes in blood levels of complement proteins also have been reported in schizophrenia [2–4]. A large study of individuals at high risk of schizophrenia found that levels of complement and coagulation proteins and particularly complement pathway inhibitors predicted which individuals subsequently developed psychosis [13]. In addition, the functional (hemolytic) activity of the complement system is consistently higher in individuals with schizophrenia vs. controls [2, 3]. Functional or hemolytic activity is measured by exposing patient serum in vitro to antigens that trigger complement cascade activity. The resulting cell lysis provides a measure of how readily the complement system can be triggered. Two small studies found increased C4 hemolytic activity in individuals with schizophrenia compared to controls [14, 15]. An unexplored question is whether variation in C4 structural genotypes contributes to complement activation in the peripheral blood of patients with schizophrenia.

Anaphylatoxins are fragments of complement proteins that are released when the proteins are activated. They are small molecules with half-lives on the order of minutes and have specific downstream receptors and functional effects [16]. C4-ana reflects C4 protein activation, which occurs in the classical and lectin complement pathways. Protease-activated receptor 1 (PAR1) and protease-activated receptor 4 (PAR4) are the only known C4-ana receptors [17]. C3-ana reflects the activity of C3-convertase, which is activated by all three complement cascades. C5-ana is released by the terminal complement cascade. Initiation of any arm of the complement cascade generally results in successive activation of subsequent pathways. The endpoint of the terminal cascade is the formation of a circular protein complex, C5b-9, or terminal complement component (TCC), that embeds itself in cell membranes and causes cell lysis (Fig. 1A). A soluble form of TCC, sTCC, can be measured in plasma and has a half-life ~1 h [18]. The literature is unclear about which complement pathways are activated in patients with schizophrenia [3]. Measuring activation products provides one way to determine which pathways are active in vivo.

Patients with schizophrenia often have evidence suggesting BBB disruption, including after traumatic brain injury, which increases the risk of schizophrenia [5, 19, 20]. A downstream effect of C4-ana binding to a PAR1/4 receptor is endothelial cell contraction and increased permeability through the endothelial cell layer [17]. Thus, C4-ana activation of PAR1/4 receptors may disrupt the BBB and contribute to neurodegeneration in individuals with schizophrenia. We hypothesized that C4-ana and thus C4 protein are increased in peripheral blood early in schizophrenia. We carried out an initial test of this hypothesis in individuals with schizophrenia with an illness duration of less than five years vs. control individuals with similar age, sex, and ethnicity.

## Materials and methods

### Recruitment of participants

We recruited 15 individuals with schizophrenia or schizoaffective disorder from academic inpatient and outpatient settings. Participants were ages 18-35, unrestricted with regard to ethnicity. Additional inclusion criteria for participants with schizophrenia included a clinical DSM-V diagnosis of schizophrenia or schizoaffective disorder, confirmation of one of these diagnoses by the Structured Clinical Interview for DSM-V (SCID-V), and initial diagnosis or initiation of antipsychotic medication within the last 5 years. Exclusion criteria included a positive urine toxicology screen or self-reported history of any of the following: current substance abuse or any use of cannabis or tobacco products, a history of bleeding disorders, excessive bleeding with previous surgery, taking blood thinners, autoimmune conditions, epilepsy, known genetic disorders, immunocompromised state, pregnancy, history of central nervous system disease, an uncontrolled medical disorder such as cancer or inability to provide informed consent. The 14 healthy control participants were matched to the same age range as the group of individuals with schizophrenia, did not meet criteria for any DSM-5 disorder by the SCID-V, and had a Distress Score of less than or equal to 6 on the Prodromal Questionnaire-Brief Version (PQ-B) [21].

### Clinical assessment

Clinical evaluation was carried out by trained doctoral-level clinical research interviewers. Participants were assessed for their capacity to participate in the research study upon interview and according to best practices [22]. All participants underwent a SCID-V interview. Controls also completed a PQ-B and patients completed a Positive and Negative Symptom Score Structured Interview (PANSS). Participants were asked about current medications (see Supplementary Material for details). Participants who completed the clinical evaluation and venipuncture were invited to return for a subsequent visit for an optional lumbar puncture.

The study protocol was approved by the Stanford University Institutional Review Board (IRB Protocol 47825). All participants gave written informed consent. Participants were compensated for study participation.

### Sample collection

All participants underwent antecubital venipuncture between 08:00 AM and 12:00 PM at the Clinical Translational Research Unit (CTRU). Blood was collected in EDTA tubes and transported on ice to the Stanford Translational Applications Service Center (TASC) for centrifugation at 1000 × *g* for 10 min at 4 °C within 30 min of collection. Immediate cooling halts protein metabolism since enzymes are optimally active at body temperature, 37 °C. The supernatant was aliquoted and snap-frozen in liquid nitrogen and stored at −80 °C until samples were analyzed. Participants underwent height and weight measurements at the time of blood draw. All collected samples were used in the analyses.

### Complement activation product measurements

Samples were shipped overnight on dry ice to Complement Laboratory of the National Jewish Medical Center (Denver, CO) where they underwent radioimmunoassay measurement of plasma and CSF C3-ana, C4-ana, and C5-ana concentrations in a CLIA-certified laboratory. sTCC, or soluble terminal complement product, concentration was measured according to manufacturer’s instructions using the Human Terminal Complement Complex ELISA kit (HK328 Hycult Biotech, Netherlands) in technical duplicates and averaged per sample.

### C4 genotype copy number determination

DNA was extracted from 0.5 to 1 mL frozen whole blood collected in EDTA tubes using the DNAeasy Blood & Tissue Kit (Qiagen, Netherlands). C4A and C4B structural polymorphism genotypes were assayed with digital-droplet PCR (ddPCR; BioRad, Pleasanton, CA) with the method of Sekar et al. [7]. Briefly, in this assay PCR probes for C4A, C4B, C4L and C4S are assayed as technical duplicates. A long-range PCR for C4S is then performed whose products are analyzed by ddPCR to determine the Gene Copy Numbers (GCN) of C4AS and C4BS, following which GCN can be computed for C4AL and C4BL. Predicted C4A and C4B expressions are then determined according to a formula [7] derived from post-mortem genotype and brain expression data:

C4A expression = (0.47 * C4AL GCN) + (0.47 * C4AS GCN) + (0.20 * C4BL GCN)

C4B expression = (1.03 * C4BL GCN) + (0.88 * C4BS GCN)

### Statistical analysis

The primary outcome was plasma C4-ana concentration in the control vs. patient groups. All other statistical tests were considered exploratory and thus were not corrected for the number of tests. Protein concentrations (C3-, C4- and C5-ana, and sTCC) and C4 GCN frequencies were not normally distributed (Shapiro–Wilk test) so that non-parametric (rank-based) tests were used for group contrasts (Mann–Whitney *U*-Test and correlational analyses Spearman’s rho). Differences between pairs of rho values were tested for significance using the appropriate modification of Fisher’s *Z*-transformation of correlation coefficients [23, 24]. All analyses were performed using JMP Pro 14.1.0 (SAS Institute, Cary, NC).

### Primary brain endothelial cell (BEC) culture

Primary human brain microvascular endothelial cells (ACBRI 376, Cell Systems, Kirkland, WA) were cultured in Complete Classic Medium with Serum and CultureBoost (4Z0-500, Cell Systems, Kirkland WA) under standard conditions (37 °C and 5% CO_2_) until a monolayer was formed. Attachment Factor (4Z0-210, Cell Systems, Kirkland, WA) and Passage Reagent Group (4Z0-800, Cell Systems, Kirkland, WA) were used for passaging according to the manufacturer’s instructions. Cells were tested for mycoplasma.

### Immunofluorescence

Primary BEC were cultured on glass chamber slides (80827, ibidi GmbH, Germany). Cells were starved in media without serum and exposed to stimulants for 30 min. Then, cells were fixed in 4% paraformaldehyde in PBS pH 7.4 for 10 min at room temperature and washed 3× with ice-cold PBS for 5 min. Cells were permeabilized with 0.1% Triton X-100 in PBS for 10 min and stained with Texas Red-X Phalloidin for 20 min at room temperature (T7471, ThermoFischer Scientific, Waltham, MA) or alternatively incubated overnight at 4 °C with primary antibody against PAR1 (sc-13503, Santa Cruz Biotechnology Inc., Dallas, TX) and PAR4 (sc-1666, Santa Cruz Biotechnology Inc., Dallas, TX). The samples were subsequently washed and incubated with secondary antibody (A21203, Life Technologies, Eugene OR) for 1 h at room temperature, and 5 min with DAPI (Sigma Aldrich, St. Louis, MO). Cells were imaged at the Stanford University Cell Sciences Imaging Facility on a Zeiss 880 confocal microscope using a ×40 objective.

### In vitro permeability assay

Transwell plates (Corning, Corning NY) were coated overnight at 4 °C with 10 μg/mL of rat tail collagen (Corning, Corning NY). Then, cells were grown on the transwell inserts until a monolayer formed. Stimulants, thrombin (T4393, Sigma Aldrich, MO) or C4-anaphylatoxin (A106, Complement Technologies, Tyler, TX) were added for 1 h, and permeability was measured according to manufacturer’s instructions (CB6929, Cell Biologics, Chicago, IL). The experiment was performed three times, each with technical duplicate wells per experimental condition.

## Results

### Characteristics of the cohort

A total of 19 controls and 16 individuals with schizophrenia enrolled in the study. Four individuals in the control group were ineligible after SCID-V assessment, and 1 control and 1 individual with schizophrenia withdrew before completing the study. Thus, 14 healthy controls and 15 participants with schizophrenia or schizoaffective disorder completed the clinical evaluation and blood sampling (Table 1). The two groups did not differ significantly with respect to age, sex distribution, or ethnicity. Together, the individuals with schizophrenia had a higher body mass index (BMI). All of the individuals with schizophrenia except one (in remission) were taking medications at the time of venipuncture (Supplementary Fig. 1). The C4-ana concentration of the medication-free individual with schizophrenia was in the upper range (688 ng/mL).

**Table 1:** Demographics of study participants.

	Controls	Schizophrenia	*p*-value
# Participants	14	15	
Median age	24	22	0.14
Age range	21–36	18–34	
Sex (% male)	64%	73%	0.46
Caucasian	9	5	
African American	2	2	
Asian	3	5	
Hispanic	1	2	
Schizophrenia	0	12	
Schizoaffective disorder	0	3	
BMI, average	24	27.5	0.01

There were no statistical differences in participant age or sex between the two groups. The average BMI (body mass index) was higher in the Schizophrenia group. Attempts were made to recruit participants from a variety of ethnic backgrounds. The majority of participants in the Schizophrenia group met the criteria for the diagnosis of Schizophrenia and 3 met DSM-V criteria for Schizoaffective disorder.

### Plasma C4-ana concentration

Concentrations of plasma C4-ana were elevated in individuals with schizophrenia compared to controls (mean ± standard deviation = 656 ± 150 ng/mL vs. 555 ± 93 ng/mL, *χ*^2^ approximation [1df] = 5.97, *p*-value = 0.015) (Fig. 1B). This difference persisted after excluding the individual with schizophrenia whose C4-ana concentration (1142 ng/mL) was an outlier value (620 ± 68 ng/mL vs. 555 ± 93 ng/mL, *χ*^2^ approximation [1df] = 5.07, *p*-value = 0.024). There are no clear relationships between plasma C4-ana concentration and BMI, fasting status or sex (Supplementary Fig. 2).

### Additional complement split products in plasma

**Fig. 2:**
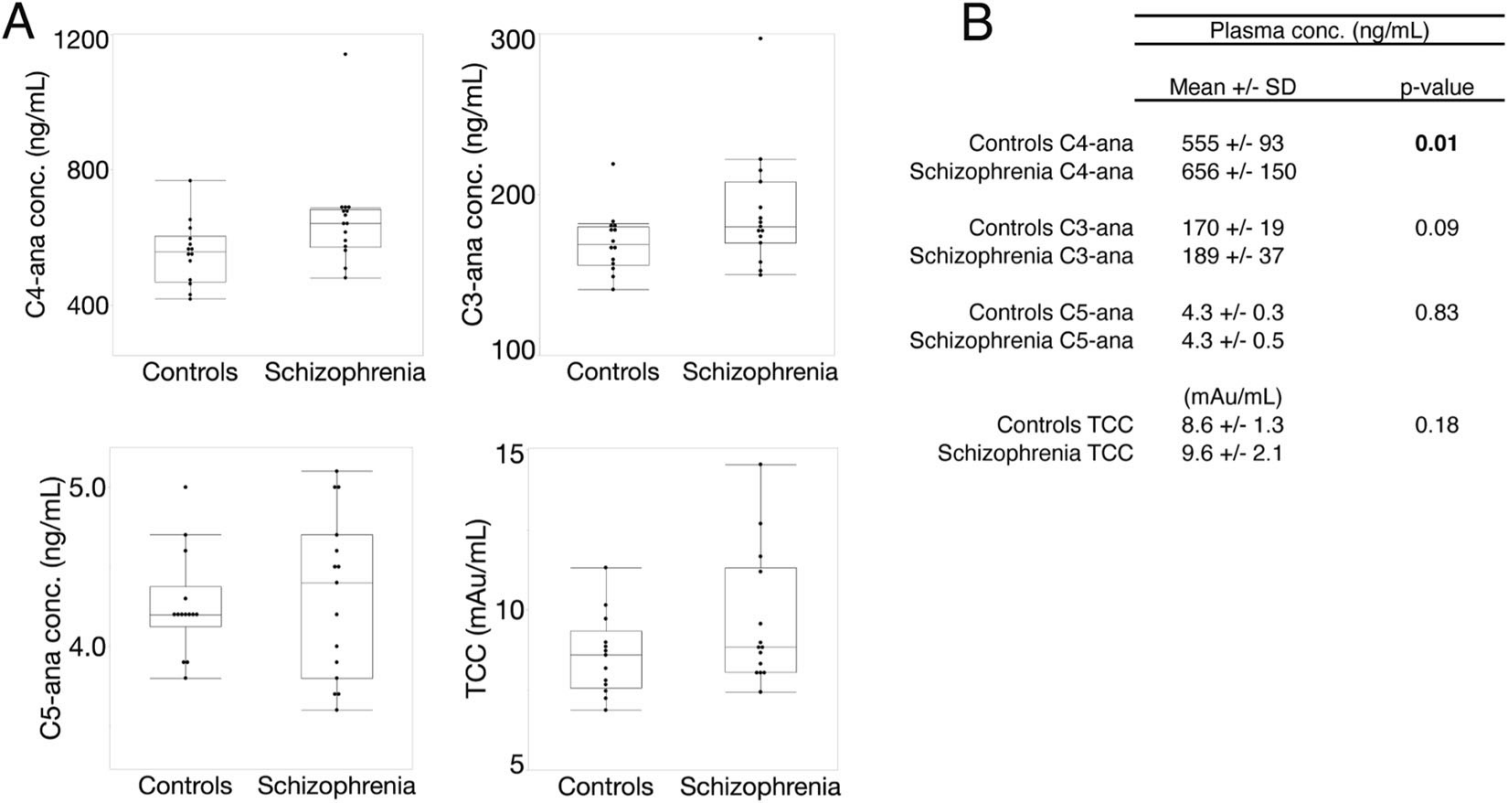
C4-ana concentration is higher in plasma from individuals with schizophrenia. **A** Concentration of C4-ana, C3-ana, and C5-ana in plasma in the whole cohort. The mean concentration of C4-ana is higher for the patient group compared to controls, *p* = 0.01 (*p* = 0.02 if the outlier is removed), as calculated by Mann-Whitney. The mean plasma levels of C3-ana, C5-ana are lower in the control group when compared to the group from individuals with schizophrenia. Detailed values and statistics are shown in the table in **B**.

Results are shown in Fig. 2. There is a trend towards increased C3-ana concentrations in individuals with schizophrenia (189 ± 37 ng/mL vs. 170 ± 19 ng/mL, *χ*^2^ approximation [1df]= 2.90, *p*-value = 0.09). C5-ana and TCC concentrations did not differ between groups.

### Complement split products in CSF

Only 5 patients and 6 controls completed lumbar puncture. Procedures are discussed and preliminary data are shown in Supplementary Fig. 3.

### C4 genotype distribution

**Fig. 3:**
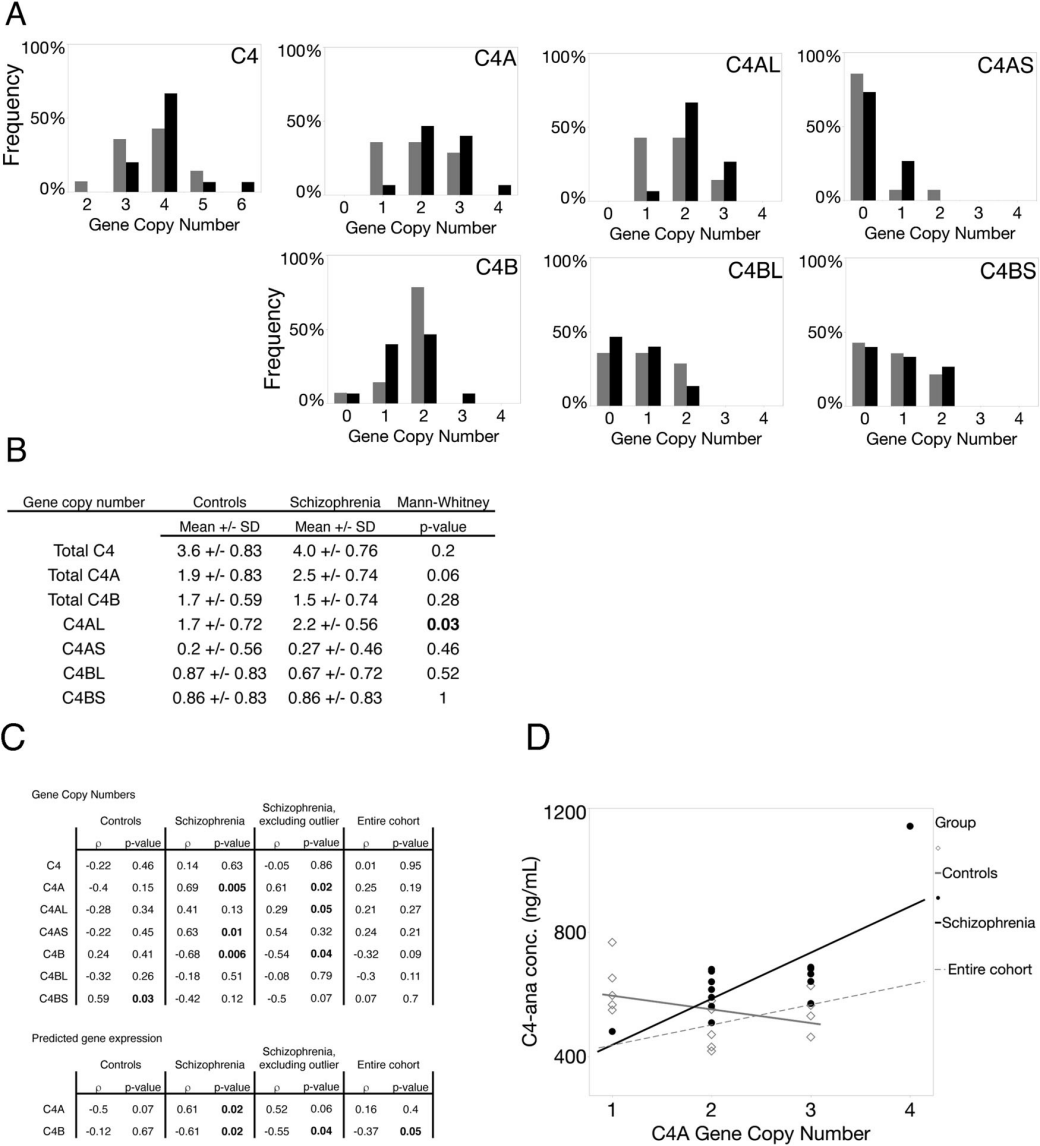
The relationship between C4-ana concentration and C4 gene copy number differs in cases vs. controls. **A** The number of C4AL, C4AS, C4BL, and C4BS genes were determined experimentally by ddPCR. The frequency of each gene variant in the control and individuals with schizophrenia groups are reported. Total C4A was determined by adding the number of C4AL and C4AS gene copies for each participant. Similarly, C4B was determined by adding the number of C4BL and C4BS gene copies for each participant. Total C4 was determined by adding the total gene copies of C4A and C4B for each participant. The frequencies of each gene copy number for participants are reported in the graphs above. The average gene copy numbers for each distribution are reported in table (**B**). In this cohort, we see that samples from individuals with schizophrenia have higher gene copy numbers of total C4A and C4AL genes compared to controls (*p*-value = 0.04 and 0.03, respectively. These *p*-values are not statistically significant if the Bonteferoni correction is applied). **C** The table shows results of Spearman’s rho correlation analyses between plasma C4-ana concentration and (i) each subset of C4 gene copy numbers (total C4, C4A, C4B, C4AL, C4AS, C4BL, C4BS); and (ii) predicted C4A and C4B gene expression in brain based on the formula of Sekar et al. **D** Plasma C4-ana concentration (*Y* axis) is plotted against total C4A (*X* axis), with regression lines shown for the entire cohort and separately for cases and controls. Cases showed a positive relationship, and controls an inverse relationship, between C4A GCN and plasma C4A GCN.

Figure 3A shows the distributions of C4 gene copy numbers (GCN) in individuals with schizophrenia and controls for the individual alleles (C4AL, C4AS, C4BL, C4BS) and for aggregate alleles (C4A, C4B, and C4). Exploratory analyses showed an increase in C4AL (*p* = 0.03) in individuals with schizophrenia and a trend for all C4A (*p* = 0.06) (Fig. 3B).

### The relationship between C4 gene copy number and plasma C4-ana levels

Figure 3C shows the correlations between plasma C4-ana and (i) GCN values (C4, C4A, C4B, C4AL, C4AS, C4BL, C4BS); and (ii) predicted C4A and C4B gene expression in the brain. In the entire cohort, the only nominally significant correlation was an inverse relationship between plasma C4-ana and predicted C4B expression (*ρ* = −0.37, *p* = 0.05). We then analyzed individuals with schizophrenia and controls separately. In individuals with schizophrenia, plasma C4-ana was positively correlated with C4A GCN (*ρ* = 0.69, *p* = 0.005); in controls, there was no correlation (*ρ* = −0.69, *p* = 0.15). The correlations in individuals with schizophrenia and in controls were significantly different based on the modified Fisher’s *Z*-transformation test for rho (*z*[difference] = −2.371, *p* = 0.018) (Fig. 3D). This difference was sensitive to the removal of the patient outlier (*p* = 0.61 in the patients, *N* = 14; *z*[difference] = −1.79, *p* = 0.073). The group-specific correlations between C4-ana and C4B GCN were in the opposite directions and were not significantly different from each other (cases: *p* = −0.68, *p* = 0.006; controls: *p* = 0.24, *p* = 0.41; *z*[difference] = 1.11, *p* = 0.27). A negative correlation between C4A and C4B GCNs is expected, based on population allele frequencies [7], as observed also in C4 GCN assays in European-, African- and Chinese-ancestry individuals [DF Levinson, unpublished data].

**Fig. 4:**
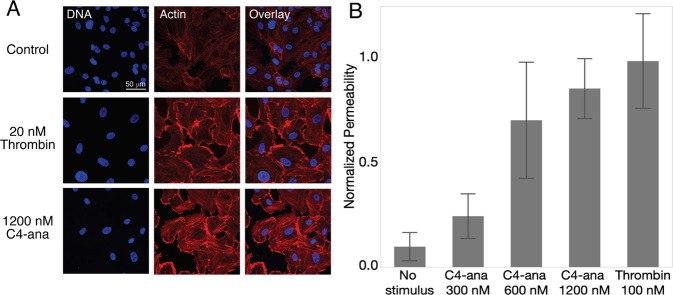
C4-ana increases the permeability of primary brain endothelial cells. **A** Brain endothelial cells stained with phalloidin and DAPI. After BEC are exposed to C4-ana, cells form actin stress fibers. **B** The permeability of the monolayer increases after exposure to increasing concentrations of C4-ana and thrombin.

### C4-ana increases in vitro permeability of brain endothelial cells in monolayer cultures

We hypothesized that increased C4-ana could have a pathological effect by binding to PAR1 and PAR4 [17] on brain endothelial cells (BEC) and causing increased permeability. We tested this hypothesis by studying monolayer BEC cultures. We confirmed that PAR1 and PAR4 receptors are present on BEC (Supplementary Fig. 4) and observed that exposure to C4-ana increased actin stress fiber formation (Fig. 4A). C4-ana, like thrombin, increased permeability of the BEC monolayer. In this assay, C4-ana was ~10× less potent than thrombin (Fig. 4B).

## Discussion

Elevated C4 gene copy number has been implicated in schizophrenia etiology by GWAS [7] and by murine models [7–9]. An allele of the complement inhibitor, *CSMD1*, is also associated with schizophrenia [3, 11, 12]. In addition, peripheral inflammation may contribute to disease pathogenesis through changes to the BBB [25]. Based on these considerations and our pilot data from individuals with schizophrenia, we hypothesized that circulating complement fragments, known to be inflammatory mediators, could be increased in this disease. To address this hypothesis, we measured the concentrations of anaphylatoxins (C3-ana, C4-ana, C5-ana) and TCC from carefully collected plasma, which provides a way to determine complement protein activation in vivo, rather than ex vivo methods that trigger activation [18].

We found significantly higher plasma C4-ana levels, but not C3-ana or C5-ana levels, in individuals with schizophrenia compared to healthy controls (Fig. 2). A previous small study of drug-naive, first-episode psychosis patients measured their TCC, C3-, and C5-ana (but not C4-ana) levels and found a trend toward elevated C3-ana. Notably, this trend was eliminated after treatment [26]. The latter result is consistent with our findings, as the majority of our participants were taking medication at the time of sampling. Two prior studies have measured in vitro activation of C4 protein, isolated from individuals with schizophrenia and controls [14, 15]. In both studies, C4 activation from individuals with schizophrenia was enhanced. This is also consistent with our results, as C4-ana is an in vivo by-product of C4 protein activation. Furthermore, several studies observed increased levels of a C4 protein inhibitor, C4-binding protein, in individuals with schizophrenia, implying an active compensatory mechanism [13, 27]. Together, these findings suggest that plasma C3-ana may play a role in acute psychosis, whereas plasma C4-ana may contribute in a more chronic manner. In general, there are no known disease associations for C4-ana, we found just one small study that showed elevated concentrations of C4-ana in multiple sclerosis [28].

Consistent with prior work [7], we found significantly increased *C4AL GNC* and a trend towards increased total *C4A GCN* in individuals with schizophrenia compared to controls (Fig. 3). Studies of post-mortem brain tissue showed higher *C4A* mRNA levels in individuals with schizophrenia compared to controls [5–7]. One study of peripheral blood mononuclear cell transcripts found increased C4A mRNA that correlated with PANSS positive symptoms factor p-scores, driven by the severity of delusions [29]. However, in two other studies, C4 mRNA levels in peripheral blood were not increased [6, 30]. In healthy individuals, peripheral blood C4A and C4B protein concentrations correlate with the C4A and C4B gene copy number but also depend on age and sex [31, 32]. The literature on the serological complement system in schizophrenia is large but characterized by small samples, variable methodological approaches, clinical diversity of cohorts, failure to assay C4A and C4B separately in most studies, and inconsistent results [2, 3, 27]. A recent meta-analysis of 10 studies of C4 protein concentration (mostly in serum) included 468 individuals with schizophrenia and 440 controls. This study did not find a difference between individuals with schizophrenia and controls, but did note a larger range of concentrations in the individuals with schizophrenia (23). Importantly, the liver is the major source of plasma C4 [33], but no studies have examined C4 mRNA in the liver in individuals with schizophrenia.

We also found a positive, significant correlation between plasma C4-ana and C4A GCN in individuals with schizophrenia (Fig. 3). A possible explanation is an enhanced susceptibility to activation of C4 protein in individuals with schizophrenia, discussed above. A mechanism for the enhancement could involve the effects of interferon-γ (INF-γ), a pro-inflammatory cytokine, that is the strongest known inducer of C4 protein expression [33, 34]. A recent meta-analysis found INF-γ to be elevated in both individuals with schizophrenia and first-episode psychosis (FEP) [35]. In a study of FEP patients who went on to a diagnosis of schizophrenia, INF-γ was elevated in FEP compared to controls and inversely correlated with the percentage of whole-brain gray matter [36]. INF-γ induction of C4 protein expression would result in more C4A-type protein in individuals with schizophrenia because they carry more copies of the C4A gene. C4A protein has differential biochemical properties and functions in synaptic pruning [9, 37] and may also be more susceptible to activation. Examining the correlation between INF-γ and C4-ana concentrations as a function of C4 GCN in both individuals with schizophrenia and controls would begin to test this hypothesis. Alternatively, unknown factors may affect C4 protein activation.

Functions of C3-ana and C5-ana are well-characterized. They bind to G protein-coupled receptors, C3aR and C5aR1, respectively, induce migration and effector function in neutrophils, mast cells, and macrophages and increase the permeability of small blood vessels [38]. In contrast, surprisingly little is known about the biology of C4-ana [39]. Although named an “anaphylatoxin,” C4-ana does not have the same mechanisms of action as C3-ana and C5-ana. The C4-ana receptor, PAR1, is expressed on neurons and astrocytes [40, 41]. Activation of PAR1 leads to apoptotic cell death in neurons [41], and to glutamate release in astrocytes producing activation of neighboring NMDA receptors on neurons [41]. These mechanisms are consistent with the glutamatergic excess found in individuals with and at risk for schizophrenia [42].

PAR1 is also expressed on leukocytes, platelets, and endothelial cells (16), including BEC [43]. Indeed, the only well-established downstream effect of C4-ana binding to a PAR1/4 receptor is endothelial cell contraction and increased permeability through the endothelial cell layer [17].

We found that the addition of C4-ana increases the permeability of a BEC monolayer (Fig. 4). A recent in vitro study demonstrated that PAR1 activation is required for lymphocyte transmigration in BEC [44]. There is evidence that suggests BBB disruption in individuals with schizophrenia [5, 19, 20]. Thus, C4-ana activation of PAR1/4 receptors may be a contributing mechanism to changing the BBB and contribute to neurodegeneration in individuals with schizophrenia.

The limitations of this study include its small sample size which limited our ability to measure group differences of several activation products. Future studies where larger sample sizes and additional makers of protein activation are needed. We were also unable to exclude two possible confounders: (i) the effect of medication and (ii) BMI on complement activation products. (i) One previous study reported an increase in C3-ana after medication treatment [26], however other studies examining the complement system did not find changes related to medication [2, 3]. (ii) We observed a trend toward a correlation between BMI and C4-ana concentrations, but only in the patient group (Supplemental Fig. 1). One study reported that BMI was strongly correlated with serum C3 and weakly with serum C4A and C4B concentrations [31, 45]. A second study reported that metabolic syndrome and waist circumference were associated with total serum C4 at baseline in a longitudinal study, with C4 level predicting the incidence of metabolic syndrome over time (C4-ana was not assayed) [31, 45]. C4-ana has not previously been studied in relation to BMI or related variables. Our data do not permit disentangling the relationships among C4-ana, antipsychotic medication, and BMI.

Further work is needed to test hypotheses arising from the current findings: that a peripheral mechanism of C4 protein activation in patients with schizophrenia interacts with increased C4 GNC and structural variants, leading to increased C4-ana. Given the small sample size of our study, larger cohorts are needed in FEP and individuals with chronic schizophrenia.

## Supplementary information

Supplementary Material
