# Supplementary material for: Increased activation product of complement 4 protein in plasma of individuals with schizophrenia
Source: Transl Psychiatry. 2021 Sep 22;11:486. doi: 10.1038/s41398-021-01583-5 (PMC8458380; doi:10.1038/s41398-021-01583-5)
Supplement: Supplementary file 1 — Supplementary Material [file 41398_2021_1583_MOESM1_ESM.docx]

SUPPLEMENTAL INFORMATION

| **# AP medications** | **Number of patients taking AP medications** | | |
| --- | --- | --- | --- |
| 0 | 1 | <1% |  |
| 1 | 8 | 53% |  |
| 2+ | 6 | 40% |  |
| List of AP meds | aripiprazole, olanzapine, risperidone, clozapine, paliperidone | | |
|  |  |  |  |
| **Taking adjunct psychotropic medications** | | |  |
| yes | 6 | 40% |  |
| no | 9 | 60% |  |
| List of adjunctive agents | citalopram, escitalopram, lithium, fluoxetine | | |

**Supplementary Figure 1: Medications taken by participants.** The majority of participants in the patient group were taking antipsychotic medication at the time of plasma collection, some on more than one antipsychotic agent and potentially an adjunctive agent. The majority of participants taking an adjunctive agent were taking a Selective Serotonin Reuptake Inhibitor (SSRI).

**Supplementary Figure 2: Exploring contributions from BMI, fasting status and sex on activation product levels. (a)** C4-ana, C3-ana and C5-ana plasma concentrations as a function of BMI for the two groups. Correlation is weak and not significant. **(b)** There is no difference between the groups when taking fasting status or **(c)** sex into account.


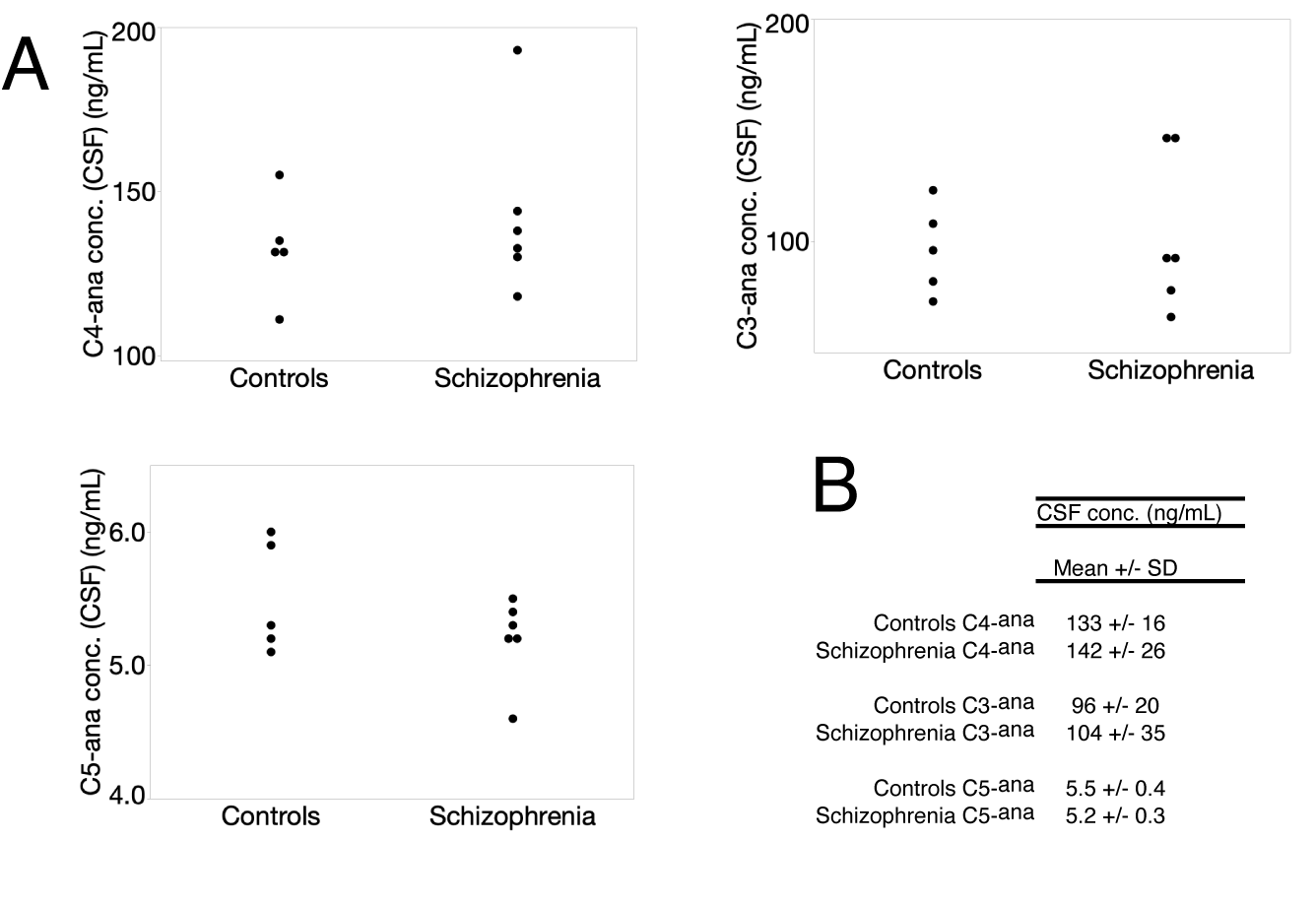


**Supplementary Figure 3: Complement split products in CSF in limited sample.** Participants who consented to CSF collection were given a subsequent appointment for a standard lumbar puncture by a neurologist under sterile conditions using an atraumatic needle when possible. Atraumatic needles have lower rates of post-LP complications (45). No post-LP complications were reported in our study. Collected CSF was transported immediately to the TASC laboratory on ice for centrifugation, as above and immediately aliquoted and frozen. Only 5 patients and 6 controls completed LP for CSF assays. Raw data points are show in the figure. No statistical analyses were applied due to limited sample size.

**
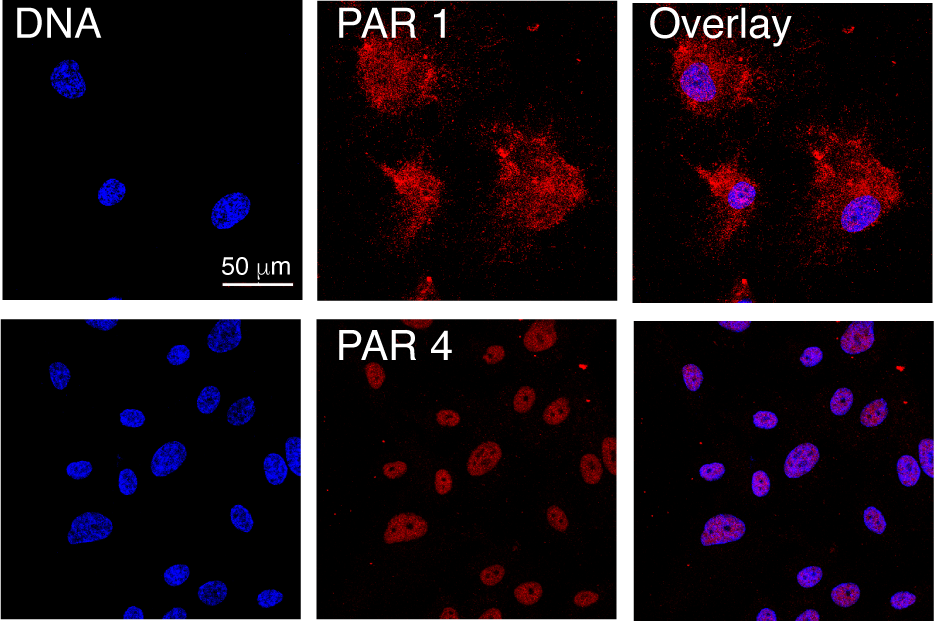
**

**Supplementary Figure 4: PAR1/4 receptor staining on BEC.** Immunofluorescent staining of BEC for DNA (DAPI) and PAR1 and PAR4. PAR1 has a cytoplasmic distribution whereas PAR4 localizes to the nucleus.
